# fastJT: An R package for robust and efficient feature selection for machine learning and genome-wide association studies

**DOI:** 10.1186/s12859-019-2869-3

**Published:** 2019-06-13

**Authors:** Jiaxing Lin, Alexander Sibley, Ivo Shterev, Andrew Nixon, Federico Innocenti, Cliburn Chan, Kouros Owzar

**Affiliations:** 1 Department of Biostatistics and Bioinformatics, Duke University, Durham, NC USA; 2 Duke Cancer Institute, Duke University Medical Center, Durham, NC USA; 3 Duke Human Vaccine Institute, Duke University Medical Center, Durham, NC USA; 4 Division of Pharmacotherapy and Experimental Therapeutics, Chapel Hill, NC USA

**Keywords:** Jonckheere-Terpstra, Linear rank statistic, Constrained inference, Robust statistic, Logarithmic complexity, Genome-wide association studies, Feature selection, Machine learning, Parallel processing

## Abstract

**Background:**

Parametric feature selection methods for machine learning and association studies based on genetic data are not robust with respect to outliers or influential observations. While rank-based, distribution-free statistics offer a robust alternative to parametric methods, their practical utility can be limited, as they demand significant computational resources when analyzing high-dimensional data. For genetic studies that seek to identify variants, the hypothesis is constrained, since it is typically assumed that the effect of the genotype on the phenotype is monotone (e.g., an additive genetic effect). Similarly, predictors for machine learning applications may have natural ordering constraints. Cross-validation for feature selection in these high-dimensional contexts necessitates highly efficient computational algorithms for the robust evaluation of many features.

**Results:**

We have developed an R extension package, fastJT, for conducting genome-wide association studies and feature selection for machine learning using the Jonckheere-Terpstra statistic for constrained hypotheses. The kernel of the package features an efficient algorithm for calculating the statistics, replacing the pairwise comparison and counting processes with a data sorting and searching procedure, reducing computational complexity from O(*n*^2^) to O(*n* log(*n*)). The computational efficiency is demonstrated through extensive benchmarking, and example applications to real data are presented.

**Conclusions:**

fastJT is an open-source R extension package, applying the Jonckheere-Terpstra statistic for robust feature selection for machine learning and association studies. The package implements an efficient algorithm which leverages internal information among the samples to avoid unnecessary computations, and incorporates shared-memory parallel programming to further boost performance on multi-core machines.

**Electronic supplementary material:**

The online version of this article (10.1186/s12859-019-2869-3) contains supplementary material, which is available to authorized users.

## Background

**Fig. 1:**
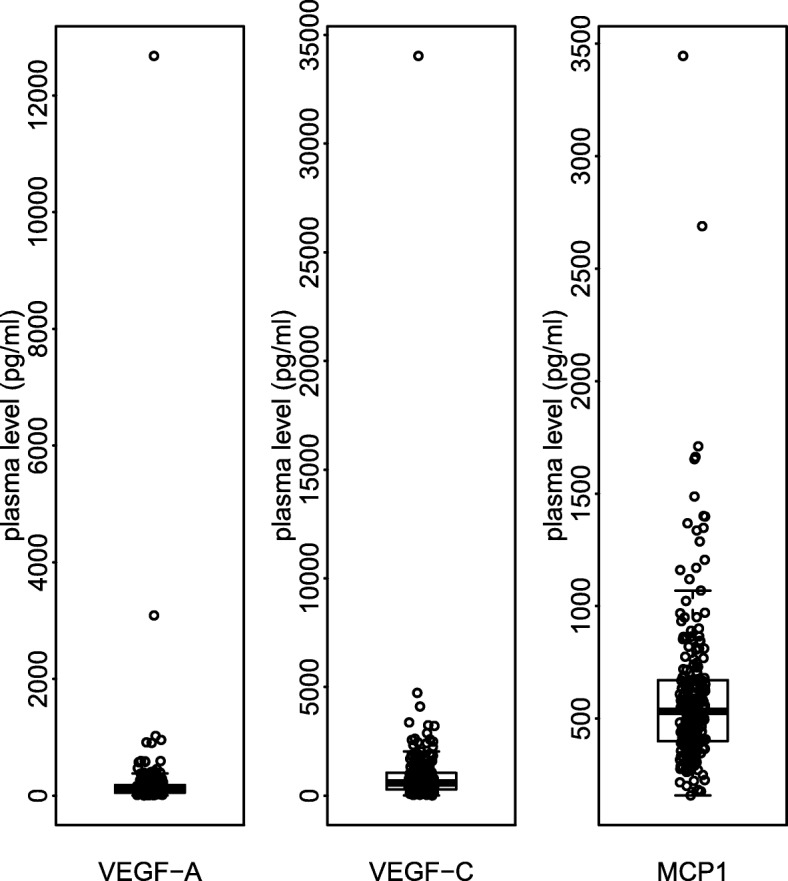
Boxplot of selected plasma protein levels for CALGB 80303 data.Box plot of VEGF-A, VEGF-C, and MCP1 plasma protein levels in CALGB 80303. The boxes indicate the 25^th^ (Q1) and 75^th^ (Q3) percentiles, with the heavy line showing the median value. Whiskers indicate max(min(plasma levels), Q1–1.5 * IQR) and min(max(plasma levels), Q3+1.5 * IQR). The circles represent individual patient plasma levels

Feature selection is a critical step in machine learning [1] and association studies based on high-dimensional genetic data. When building a model, attempting to use all available features can perform as poorly as random guessing [2]. The key to building a generalizable model is a robust feature selection procedure that minimizes the inclusion of noisy features due to the presence of a few outliers or influential observations. Similarly, a robust feature selection procedure protects against over-inflation of the significance of a null variant. To illustrate this point, we consider an example from a recently published genome-wide association study (GWAS) which identified common variants associated with baseline circulating protein levels in advanced pancreatic cancer patients [3]. Figure 1 illustrates the presence of extreme outliers for three markers from this GWAS. These examples underscore the need for inferential methods which are robust to such potentially influential observations.

Both parametric and nonparametric methods for feature selection are available. Parametric tests are more widely adopted for feature selection, as they are generally faster and, under certain assumptions, have greater power. Furthermore, the parameter estimates of the parametric models often have an intuitive interpretation. However, the statistical validity of parametric methods is dependent on distributional assumptions, for example normality or homogeneity of the variability of the observations. Conversely, nonparametric tests, also known as distribution-free tests, do not require assumptions about the distribution of the data and are, unlike their parametric counterparts, robust to outliers and influential points. For a general comparison of parametric versus nonparametric methods see [4].

Commonly employed nonparametric tests include the Wilcoxon rank-sum (Wilcoxon-Mann-Whitney) [5] and the Kruskal-Wallis [6] tests, which are nonparametric counterparts of the two-sample t-test and the analysis of variance (ANOVA) model, respectively. Feature selection theory based on the Wilcoxon rank-sum test has been proposed to study lung and prostate cancers [7]. For ordered alternatives, the Jonckheere-Terpstra (JT) test [8, 9] is a nonparametric counterpart to the simple linear regression model.

The JT test is used to assess whether a quantitative trait is associated with an ordinal feature. For example, the association between ammonia levels and the severity of hepatic encephalopathy [10], and the association of abnormal MRI findings with bone-marrow-related disease [11]. More recently, studies have used the JT test to investigate the association between single nucleotide polymorphisms (SNPs) in human genes and quantitative phenotypes [12–18]. However, in these cases the use of the JT test for feature selection in genomic studies is limited to small data sets, examining only a few candidate SNPs. Feature selection for high-dimensional problems, for example the analysis of SNPs genome-wide, or mapping expression quantitative trait loci (eQTL), using the JT test is computationally expensive and often infeasible, since the computational complexity of the JT test is quadratic in the number of samples. Further, robust feature selection for machine learning involves not only the application of the JT test to high-dimensional data, but also cross-validation, to avoid overfitting. Cross-validation requires repeating the JT test on different subsets of the data, demanding significant computational resources.

In this paper, we present fastJT, an open source R [19] package featuring an efficient implementation of the JT test that is capable of computing statistics across multiple features and quantitative traits. The naive/brute force implementation of the JT test has computational complexity of O(*n*^2^), where *n* is the number of samples. By replacing the pairwise comparison of quantitative traits values with a sorting and searching algorithm, we are able to achieve a computational complexity of O(*n* log(*n*)). This improvement makes the computing of large numbers of rank-based, distribution-free statistics possible within reasonable computational time. fastJT also provides a function for cross-validation-based feature selection using the JT test. In the following sections, we provide a detailed explanation of how the O(*n* log(*n*)) complexity is achieved, a description of the new algorithm, and a series of benchmarking examples for selecting top features for GWAS and machine learning. We demonstrate the application of the algorithm to real data, and conclude with a discussion of the benefits and limitations of this implementation.

## Methods

### Computational complexity

The JT test assesses the association between an ordinal feature and a quantitative trait. The latter could be a clinical outcome (e.g., blood pressure) or a biomarker (e.g., protein or mRNA level). The JT test compares the values of the quantitative trait between samples from the different ordinal groups. The resulting test statistic is composed of a weighted sum of these comparisons.

The contribution to the statistic from any pair of ordinal groups is the Mann-Whitney count [5], given by [20]

1 $$ U_{k l} = \sum_{i=1}^{n_{k}} \sum_{j=1}^{n_{l}} \phi(y_{ik},y_{jl}).  $$

Here, *k* and *l* are the indices for the ordinal groups being compared (assume *k*<*l*). The numbers of samples in each group are *n*_*k*_ and *n*_*l*_, and *y*_*ik*_ and *y*_*jl*_ represent the values of the quantitative trait for the *i*^*th*^ and *j*^*th*^ members of the groups, respectively. The function *ϕ*(*a*,*b*) has a value 1, 0.5, or 0, if *a* is less than, equal to, or greater than *b*, respectively. This group-wise comparison is carried out for each pairing of *t* ordinal groups, resulting in a total of *t*(*t*−1)/2 between-group comparisons. Note that the ordinal categories being compared are predetermined by the experimental design, as opposed to being based on the observed data (Section 6.2 in [20]). In association studies based on genetic data, as in the case of a linear regression, we treat the SNP as a non-random predictor. The full statistic for the JT test is then the summation of the values of *U*_*kl*_ for each possible *k* and *l* combination,

2 $$ \mathbf{J} = \sum_{k=1}^{t-1} \sum_{l=k+1}^{t} U_{kl}.  $$

Note that computing the standardized version of the JT statistic requires the calculation of the expected value and the variance of **J** (Eq. 6.19 in [20]). The computational overhead of these additional steps is relatively small (O(1)), so the overall computational complexity is dominated by the calculation of **J**, which has a cost of $\mathrm {O}(\sum _{k=1}^{t-1} \sum _{l=k+1}^{t} n_{k} n_{l})$ for this simple pairwise comparison scheme.

The number of pairwise comparisons required is greatest when the ordinal groups are of equal size. Since the number of ordinal categories, (and therefore the number of between-group comparisons), is a predetermined constant, the total computational complexity will be $\mathrm {O}(\bar {n}^{2})$, where $\bar {n}=n/t$, (see “Appendix: Computational Complexity” for detailed derivations), i.e., quadratic in the number of samples. When analyzing multiple features and quantitative traits, this calculation must be conducted *m*×*p* times, where *m* is the number of features and *p* is the number of quantitative traits. This can become very expensive for some applications, e.g., a GWAS, which might involve dozens of biomarkers and thousands or millions of SNPs. Therefore, a faster algorithm for computing JT test statistics is highly desirable.

One way to reduce the computational complexity is to sort the values of the quantitative trait prior to calculation of the statistic, to minimize unnecessary and repeated comparisons. This comes at the cost of adding a preliminary sorting step. In general, a sorting algorithm can be used that has (as an upper bound) O(*n* log(*n*) complexity. After sorting, many unnecessary comparisons can be avoided (the details of this are presented in the “Algorithm and implementation” section below). It can be shown that the computational cost for obtaining the JT test statistic for sorted quantitative traits (ignoring, for a moment, the cost of sorting) is $\mathrm {O}(\bar {n})$, i.e., linear in the number of samples. It can further be shown that the computational complexity of the sorting step is $\mathrm {O}\left (\bar {n}\log (\bar {n})\right)$ (see “Appendix: Computational Complexity” for detailed derivation), where $\bar {n}$ could be interpreted as the mean number of samples per ordinal group. Thus the overall cost will be dominated by the sorting process. For relatively large samples, (i.e., $\bar {n} > 50$), this is a significant improvement over the quadratic complexity of the naive, pairwise comparisons. If the sample size is very close to the number of subgroups, or if the samples are very sparse, then the improvements in performance may not be as dramatic.

### Algorithm and implementation

The essence of the JT test is to count the number of observations in one ordinal group that have smaller, larger, or equal values of the quantitative trait compared to the observations in higher ordinals. Recall, however, that for *k*<*l*, counts of *y*_*ik*_>*y*_*jl*_ are weighted by zero. Therefore, we can improve computational efficiency by avoiding such noncontributing comparisons. In that case, Eqs. 1 and 2 can be written as

$$\mathbf{J} = \sum_{k=1}^{t-1} \sum_{l=k+1}^{t} \sum_{i=1}^{n_{k}} \sum_{j s.t. y_{jl} \ge y_{ik}} \phi(y_{ik}, y_{jl}). $$

Further gains can be made if we increment the indices *i* and *j* in order of ascending values of the quantitative trait for each ordinal group. In this case, no further comparisons are needed once *y*_*ik*_ is strictly less than *y*_*jl*_. The additional contribution to the statistic is equal to the number of remaining members of group *l* such that *y*_*jl*_>*y*_*ik*_.

The algorithm below demonstrates how this improved counting process can be implemented. Initially, the values of *y*_*ik*_ and *y*_*jl*_ for groups *k* and *l* are sorted in ascending order into vectors *Y*_1_ and *Y*_2_, respectively. Then the computation is conducted as follows:


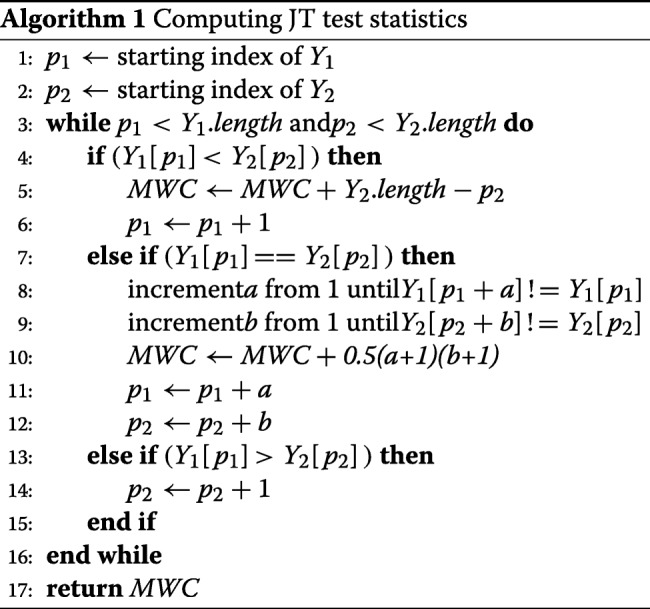


The pointers *p*_1_ and *p*_2_ proceed along the vectors *Y*_1_ and *Y*_2_, moving one or, in the case of equal values, multiple positions at a time, thereby accounting for all comparisons while requiring only a single pass over the two vectors. Therefore the computational complexity of computing the Mann-Whitney count *U*_*kl*_ is *O*(*n*_*k*_+*n*_*l*_), i.e., linear in the number of samples involved. The overall cost of computing **J** is a summation of the above calculations for all *t*(*t*−1)/2 between-group comparisons. Since *t* is predetermined, the computation complexity is $\mathrm {O}(\bar {n})$, remaining linear in the number of samples.

This algorithm is implemented in the R [19] extension package fastJT [21], primarily coded in C++ and ported to the R platform using Rcpp [22]. This package is designed to compute the JT test statistics for large numbers of ordinal features and quantitative traits for conducting feature selection for machine learning or GWAS. The package provides functions for k-fold cross-validation of the feature selection process. Incorporation of the C++ library OpenMP [23] enables simultaneous testing of multiple phenotypes, as for example in the case of eQTL analyses, improving performance for analyzing large data sets.

### Example applications to real data

Here we demonstrate the use of fastJT in a machine learning application to real-world data. We illustrate a practical application of our package in the context of conducting cross-validated robust feature selection for building a model to predict baseline circulating protein levels for the three biomarkers, VEGF-A, VEGF-C and MCP1, shown in Fig. 1. These analyses are based on genome-wide genotyping and plasma protein marker data from CALGB 80303, a randomized, placebo-controlled, double-blind, phase III study of bevacizumab plus gemcitabine in advanced pancreatic adenocarcinoma patients [3, 24–26]. For these analyses, we use data from 216 CALGB 80303 patients who are estimated to be genetically European and have baseline circulating protein level data available. The number of SNPs in this analysis is 484,523. Additional information about these data, including quality control methods used for the genotype and protein marker assays, are provided in the four previously referenced papers. The genotyping data from CALGB 80303 can be downloaded from the database of Genotypes and Phenotypes (dbGaP) through study accession phs000250.v1.p1. The protein marker data are provided as part of the supplemental information from Innocenti et al. [3].

For each marker, the predictive model is trained based on a two-layer cross-validation approach as described by Simon et al. [27]. The outer layer utilizes leave-one-out cross-validation for robust feature selection using fastJT, while the inner layer uses 10-fold cross-validation to tune an elastic net model using the cv.glmnet function from the glmnet [28] package. In the outer layer, the data from a single patient are removed, and the JT test is applied to the data from the 216−1=215 remaining patients. The resulting *P*-values are used to select the top 10 features. In the inner layer, the elastic model is tuned using data from the 215 patients on the basis of the features selected from the outer layer. The resulting model is used to predict the plasma protein level for the patient which was initially removed, and this process is repeated for all patients. The cross-validated predicted levels are then compared to the corresponding observed levels using *R*^2^ values and scatter plots. See Additional file 1: Figure S1 for a schematic of this process.

## Results

In this section, we provide benchmarks to demonstrate how the computational cost of our algorithm is related to the number of samples and the dimensions of the data. We also assess the statistical operating characteristics of our method in the presence of outliers. Benchmarks for parallel analyses are also reported, to explore the cost of the overhead required for multiple threading. Benchmarking was conducted on an AMD Opteron(tm) 6180 SE CPU running the Debian 4.9.130-2 AMD64 GNU/Linux operating system. We also demonstrate a real-world application of our algorithm using data from the CALGB 80303 GWAS.

### Simulation and benchmarking

We begin by benchmarking the performance of fastJT in the context of feature selection for a simulated machine learning analysis, looking at the computational costs of k-fold cross-validation. A single quantitative trait (*p*=1) is sampled from *N*(0,1), a normal distribution with mean 0 and variance 1. The features (*m*=1,000,000) are each sampled from *B*(2,0.5), a binomial distribution with size parameter 2 and probability parameter 0.5. These represent counts of variant alleles (0, 1 or 2) from genotyping biallelic SNPs. The traits and features are simulated for *n*=1,000 observations, and each simulation is replicated *B*=100 times.

**Fig. 2:**
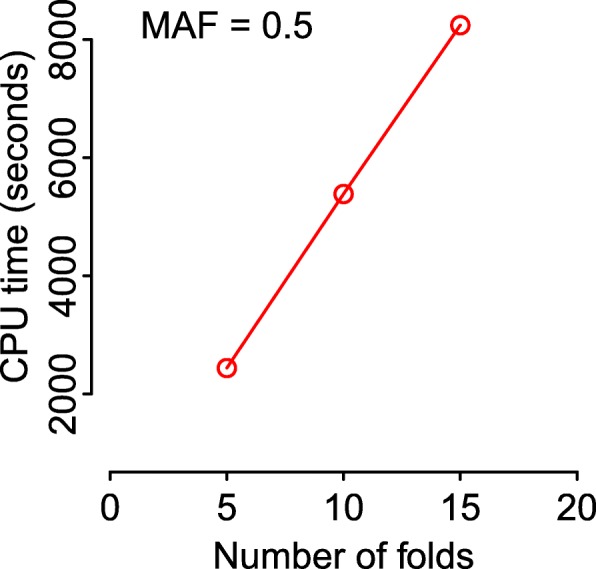
Cross-validation CPU times. CPU times for computing standardized JT test statistics using cross-validation with different numbers of folds, *k*, based on *n*=1,000 samples with *m*=1,000,000 features and *p*=1 quantitative trait. Each reported time is the mean of *B*=100 simulation replicates

Figure 2 shows the CPU times for feature selection corresponding to an analysis using the full training set, and *k*=5,10 and 15 fold cross-validation.

The remaining simulation studies are conducted within the context of an eQTL analysis. In the first study, we benchmark the effects of varying the sample size, *n*, number of features, *m*, or number of traits, *p*, on execution time. In the second study, we benchmark the performance gained from using the OpenMP implementation of our method. In the third study, we assess the robustness of our approach in presence of outliers, as compared to a parametric method.

For the first two studies, each of the *p* quantitative traits are sampled from *N*(0,1), a normal distribution with mean 0 and variance 1, and the genotypes of the *m* SNPs are sampled from a binomial distribution with size 2 and probability MAF where MAF, the relative minor allele frequency, is 0.5. For the final study, which involves outliers, the quantitative traits for samples simulated as having two minor alleles are drawn from a mixture distribution consisting of components *N*(0,1) (standard) and *N*(8,1) (outlier), with mixture probability *π* ∈[0,0.03], the proportion of the observations drawn from the outlier component. The simulations are repeated for MAF equal to 0.2,0.3,0.4, and 0.5.

**Fig. 3:**
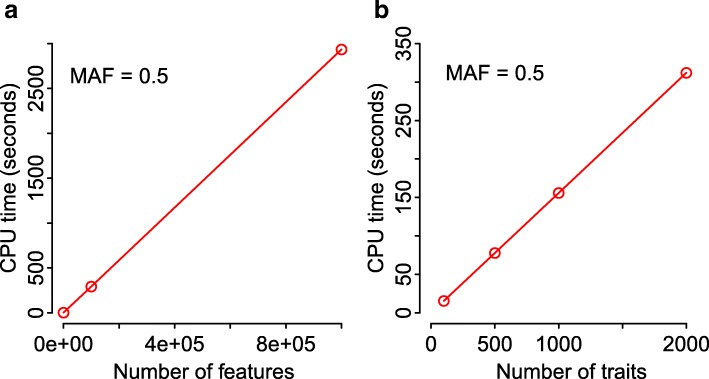
CPU times for varying numbers of SNPs and traits. **a**: CPU times for computing standardized JT test statistics for different numbers of SNPs, *m*, with a fixed number of traits (*p*=50) and samples (*n*=1,000) using 8 threads. **b**: CPU times for computing standardized JT test statistics for different numbers of traits, *p*, with a fixed number of SNPs (*m*=1,000) and samples (*n*=1,000). Reported time for panel (**a**) is the mean of *B*=10 simulation replicates. Reported time for panel (**b**) is the mean of *B*=100 simulation replicates

We first consider the effect of varying the number of features or traits. Figure 3a shows the CPU times for simulations with varying numbers of SNPs, *m*, and a fixed number of traits (*p*=1,000) and samples (*n*=1,000). Figure 3b shows results for a similar study, but instead varies the number of traits, *p*, while holding the number of SNPs fixed (*m*=1,000).

**Fig. 4:**
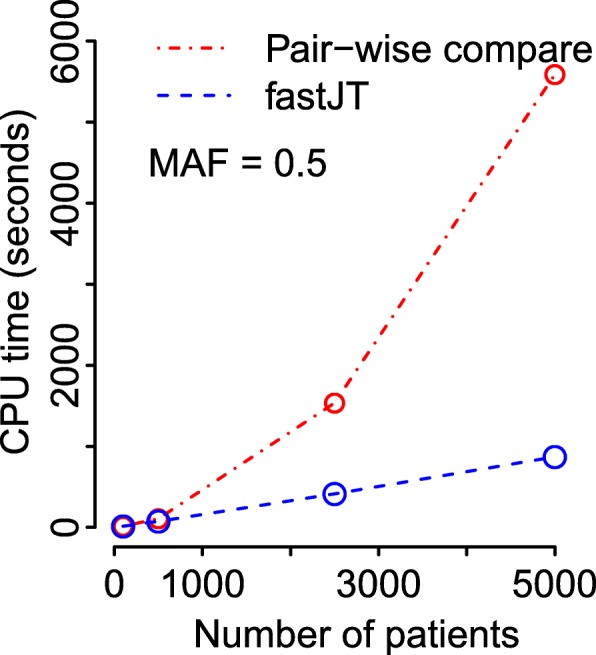
CPU times for varying numbers of samples. CPU times for fastJT and an implementation of the JT test using (unsorted) pairwise comparisons. Results are shown for different numbers of samples (*n*), with a fixed number of SNPs (*m*=1,000) and traits (*p*=1,000). Each reported time is the mean of *B*=100 simulation replicates

We next examine the computational cost of increasing the sample size, comparing two different implementations of the JT test. The first implementation is fastJT, using the algorithm described above. The second is a “naive” implementation of the test, which does not include the sorting step. This algorithm therefore requires exhaustive pairwise comparisons. Figure 4 shows the CPU times based on a fixed number of features (*m*=1,000) and traits (*p*=1,000), with a varying number of samples (*n*). Each reported time is the mean of *B*=100 simulation replicates.

Specifically, we observe that if the number of samples is increased from 100 to 5000, the corresponding CPU time for fastJT is increased from 13.8 to 870.2s. The corresponding times based on the naive pairwise comparison algorithm are 14.7s and 5589.0s, respectively.

Beyond improving algorithmic efficiency, computation time for investigating multiple features and quantitative traits can be reduced by processing separate tests in parallel. However, this approach can incur additional overhead due to the cost of passing data to, and retrieving results from, the processing cores.

**Fig. 5:**
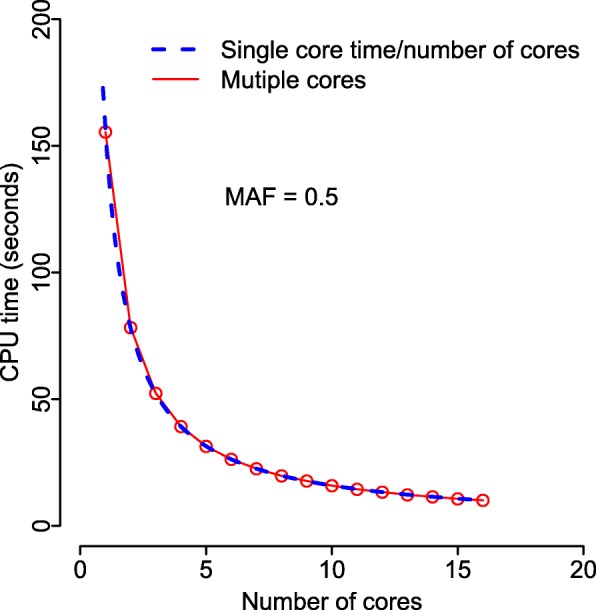
CPU times for varying numbers of processing cores. CPU times for different numbers of processing cores, with a fixed number of SNPs (*m*=1,000), traits (*p*=1,000), and samples (*n*=1,000). The dashed line gives the single core CPU time divided by the number of cores. Each reported time is the mean of *B*=100 simulation replicates

Figure 5 shows the elapsed CPU times for for a fixed number of traits (*p*=1,000), SNPs (*m*=1,000), and samples (*n*=1,000), when using different numbers of parallel processing cores. The red dots represent the CPU times for the multi-core OpenMP implementation. For comparison, the dashed curve shows the single core CPU time divided by the number of cores. This represents the idealized parallel processing time in which there is no overhead for passing data among cores.

Having explored the computational performance of our algorithm empirically, we next examine its statistical performance in the presence of outliers. This is shown in the context of inference for a single feature/quantitative trait pair (*m*=1, *p*=1) in *n*=500 samples. For this illustration, we simulate the genotypes for a variety of MAFs, and simulate the quantitative trait to include a small proportion of outliers, as described above. The statistical performance of the fastJT algorithm is compared to that of the simple linear regression model. The simple linear regression model is a parametric counterpart to the JT test for testing the association of ordinal and a quantitative variables, using the genotype as a numeric predictor of the marker level. We consider the rejection rate at the nominal 0.05 level (i.e., we call the feature to be significant if the corresponding asymptotic *P*-value is less than 0.05).

**Fig. 6:**
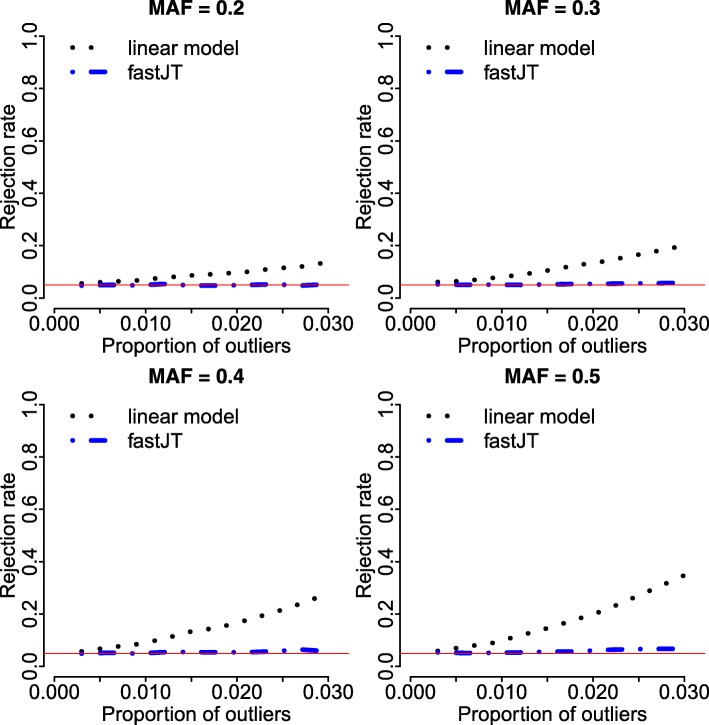
Empirical rejection rates in the presence of outliers. Empirical rejection rates for the linear regression model and the JT test, for data simulated with *n*=500 samples for varying levels of MAFs and varying proportions of outliers, *π*. The red solid line indicates the nominal rate of 0.05. Each reported rate is based on *B*=10,000 simulation replicates

Figure 6 shows the rejection rates for the linear model, using the lm function in R, and the JT test, as implemented in fastJT, for varying levels of *π*, the proportion of the observations drawn from the outlier distribution.

### Example application to real data

**Fig. 7:**
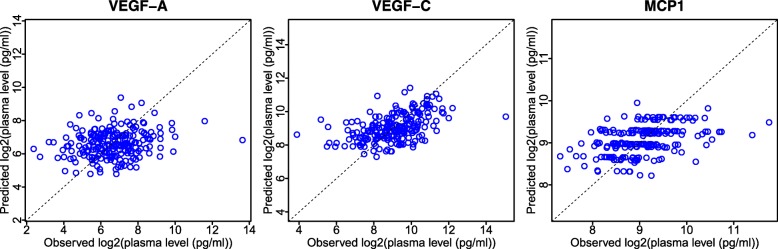
Machine learning prediction of plasma levels in CALGB 80303. Comparison of observed and predicted plasma protein levels of VEGF-A, VEGF-C, and MCP1. The machine learning model is built based on the top 100 SNPs selected by fastJT, and trained using the glmnet package

In Figure 7, we show scatter plots of the predicted versus observed marker levels for VEGF-A, VEGF-C and MCP1 from the CALGB 80303 GWAS. The analysis is conducted on an AMD Opteron(tm) 6386 SE CPU running the Ubuntu 18.04.1 LTS GNU/Linux operating system. The completion of the entire analysis, including the robust feature selection step using fastJT, is 2.51 hours. Note that this process includes executing 216 leave-one-out cross-validation replicates, each of which consists of a GWAS of three protein markers, based on 215 patients and 484,523 SNPs. The predicted and observed circulating protein levels for VEGF-A, VEGF-C and MCP1 have *R*^2^ values of 0.047, 0.24 and 0.19, respectively.

As another example application, the fastJT algorithm is used by Innocenti et al. [3] to conduct an eQTL analysis of data from CALGB 80303 [24] and 80203 [29].

## Discussion

These benchmarks provide a practical demonstration of how the computational cost of our JT test algorithm is related to the number of samples and the dimensions of the data. These simulations and results are completely reproducible using the code included with this manuscript as Additional files 2, 3, 4 and 5. In addition to the evaluations shown here, we also confirm the accuracy of the calculation against known results based on example 6.2 of [20]. The statistics calculated by our fastJT package match the published results. The code used for this verification is provided as Additional file 6.

While the computational costs given here would be greatest if the simulated samples were evenly distributed across the ordinal groups, i.e., if the simulated counts of the variant alleles (0, 1 or 2) occured in proportions of 1/3, 1/3 and 1/3, a binomial distribution with probability parameter 0.5, giving proportions of 1/4, 1/2, and 1/4, represents the worst case under Hardy-Weinberg.

Figure 2 shows that the CPU times grow linearly with the number of folds, since each cross-validation is a repeat of the JT test (excluding a subset of samples). The CPU times also grow linearly with the numbers of features and traits (Fig. 3), when the other parameters are fixed, since the JT test is conducted for all possible combinations of features and traits.

When the sample size is increased (Fig. 4), a 50-fold increase in sample size corresponds to a similar relative increase, about 50-fold, in execution time for fastJT, while the naive pairwise comparison algorithm shows a more than 380-fold increase.

The limiting factor in the performance of our algorithm is the efficiency of the sorting algorithm. The computational complexity of $\mathrm {O}(\bar {n}\log (\bar {n}))$ is the worst case. In our simulations, as shown in Fig. 4, the computational complexity is $\mathrm {O}(c\times \bar {n}\log (\bar {n}))$, where *c* is a very small factor, thanks to the efficient sorting algorithm implemented in C++ STL. Ultimately, the optimal choice of sorting algorithm depends on the data type.

For application of the JT test, the trait is assumed to be continuous while the feature is assumed to be ordinal. Note that by the nature of the Jonckheere-Terpstra test, our approach is not applicable in the case when both the trait and feature are continuous, except when such features can reasonably be binned into a manageable set of ordinal values. The algorithm also lacks support for selection of interaction effects. In the case of overlapping or correlated features, additional preprocessing, e.g., LD pruning, is required to prevent the selection of redundant features.

When leveraging OpenMP for multi-core processing, as shown in Fig. 5, the observed times match closely with the theoretical ideal, indicating that the overhead of performing the multi-core computation is negligible.

Finally, in Fig. 6 we observe that for small proportions of outliers, the empirical rejection rate for the parametric approach exceeds the nominal rate of 0.05. The JT test, while more computationally demanding compared to the parametric approach, as expected, is robust to the presence of outliers.

In addition to benchmarking on simulated data, we use our package to to build prediction models for three circulating protein markers from the CALGB 80303 GWAS. This application of the algorithm for robust feature selection produces predictions of the observed marker levels within a few of hours using a desktop CPU. Because the predictions are based leave-one-out feature selection, processing time is dominated by the number of patients, rather than the number of traits being predicted.

The *R*^2^ values between the observed and cross-validated predicted levels of circulating VEGF-A, VEGF-C and MCP1 (Fig. 7), which are 0.047, 0.24 and 0.19, respectively, provide estimates of the proportion of the variability in the marker levels that can be attributed to common genetic variation. We note that if one skips the outer layer of the cross-validation, by selecting the top ten features based on data from all 216 patients, and then reusing the same set of features for each of the 216 leave-one-out cross-validation replicates, the estimated *R*^2^ values are 0.38, 0.31 and 0.20 for VEGF-A, VEGF-C and MCP1, respectively. The risk of producing potentially over-optimistic results, when the feature selection process is not cross-validated, is well known [27, 30]. The main objective of this example is not to carry out a full analysis of the estimated proportion of variability of these protein markers explained by common genetic variation but rather to illustrate a practical application of our package for properly cross-validated robust feature selection.

The key feature of the proposed algorithm is that it leverages the sorting of sample values to minimize unnecessary comparisons when calculating the statistics. The gain in performance of this algorithm over pairwise comparisons is substantial (Fig. 4), and this improvement is magnified as the sample size increases. The JT method shows markedly less inflation of the empirical rejection rate in the presence of outliers than the method of linear models. While these results are shown in the context of feature selection for machine learning and trait-SNP associations, our approach can be applied to a wide variety of statistical problems requiring large-scale feature selection.

## Conclusion

This paper introduces the R extension package fastJT for robust and efficient feature selection for machine learning and genome-wide association studies with multiple quantitative phenotypes. The package employs an efficient algorithm which uses sorting to avoid unnecessary and redundant computations. This algorithm achieves a reduction in computational complexity to $\mathrm {O}(\bar {n}\log (\bar {n}))$, compared to O(*n*^2^) for the naive approach. The JT test is robust in the presence of outliers when compared to a parametric linear regression test. fastJT is released under a public license and provides ample documentation, including a vignette.

## Appendix: Computational Complexity

The core of the JT test statistic is the the Mann-Whitney count [5]:

$$U_{kl} = \Sigma_{i=1}^{n_{k}}\Sigma_{j=1}^{n_{l}} \phi(y_{ik},y_{jl}). $$

Here, *k* and *l* are the ranked indices for the ordinal groups being compared (assume *k*<*l*). The numbers of samples in each group are *n*_*k*_ and *n*_*l*_, and *y*_*ik*_ and *y*_*jl*_ represent the values of the quantitative trait for the *i*^*th*^ and *j*^*th*^ members of the groups, respectively. Then the JT test statistic is the summation of the Mann-Whitney counts across all pairings of the ordinal categories

$$\mathbf{J} = \sum_{k=1}^{t-1} \sum_{l=k+1}^{t} U_{kl}, $$

where *t* is the number of categories. The overall computational complexity of the above process has a cost (in terms of elementary operations) of

$$\sum_{k=1}^{t-1} \sum_{l=k+1}^{t} n_{k} n_{l}. $$

In the worst-case scenario, all groups have size $\bar {n}=n/t$. The total computation cost can then be given as

$$\frac{t(t-1)}{2} \bar{n}^{2}. $$

Since the number of categories, *t*, is a predefined constant, the computational complexity, in big O notation, is:

$$\mathrm{O}\left(\bar{n}^{2}\right). $$

That is to say, the computational cost for a naive implementation of the JT test statistics is quadratic in the number of samples.

In the fastJT package, the computation of the statistics is carried out in two parts, sorting and counting. After sorting the quantitative trait, the Mann-Whitney count is carried out by conducting a searching and counting process, shown in the “Algorithm and implementation” section to have a computational complexity of $\mathrm {O}(\bar {n})$. For this implementation of the statistic, the computational cost, in terms of the total number of elementary operations, is:

$$t \bar{n}\log(\bar{n}) + \mathrm{O}(\bar{n})= \mathrm{O}(\text{max} (t \bar{n}\log(\bar{n}), \bar{n})). $$

The logarithmic term of the cost is incurred from sorting the quantitative trait values for the samples in each of the ordinal groups. The linear term accounts for the searching and counting process. Thus, the sorting process dominates the overall cost of computation, so the computational complexity for computing the JT statistic in fastJT is $\mathrm {O}(t\bar {n} \log (\bar {n}))$, or essentially $\mathrm {O}(\bar {n} \log (\bar {n}))$ for a fixed number of ordinal categories.

## Additional files

Additional file 1 A figure of the schematic for two-layer cross-validation machine learning model. (PDF 203 kb)

Additional file 2 An R script verifying the accuracy of the fastJT package results compared to the examples in the literature. (R 1 kb)

Additional file 3 An R script performing the benchmarking of the fastJT algorithm reported in this paper. (R 3 kb)

Additional file 4 An R script for comparing the empirical rejection rates between the JT method and the linear regression method. (R 2 kb)

Additional file 5 An R script for producing the figures presented in this paper. (R 6 kb)

Additional file 6 An R script demonstrating using the fastJT package for feature selection for machine learning based on data from CALGB 80303. (R 8 kb)

## Abbreviations

ANOVA: analysis of variance
eQTL: expression quantitative trait loci
GWAS: genome-wide association studies
JT: Jonckheere-Terpstra
MAF: minor allele frequency
SNP: single nucleotide polymorphism
