# Supplementary material for: fastJT: An R package for robust and efficient feature selection for machine learning and genome-wide association studies
Source: BMC Bioinformatics. 2019 Jun 13;20:333. doi: 10.1186/s12859-019-2869-3 (PMC6567636; doi:10.1186/s12859-019-2869-3)
Supplement: Supplementary file 1 — A figure of the schematic for two-layer cross-validation machine learning model. (PDF 203 kb) [file 12859_2019_2869_MOESM1_ESM.pdf]

Schematic for two-layer cross-validation machine learning model.

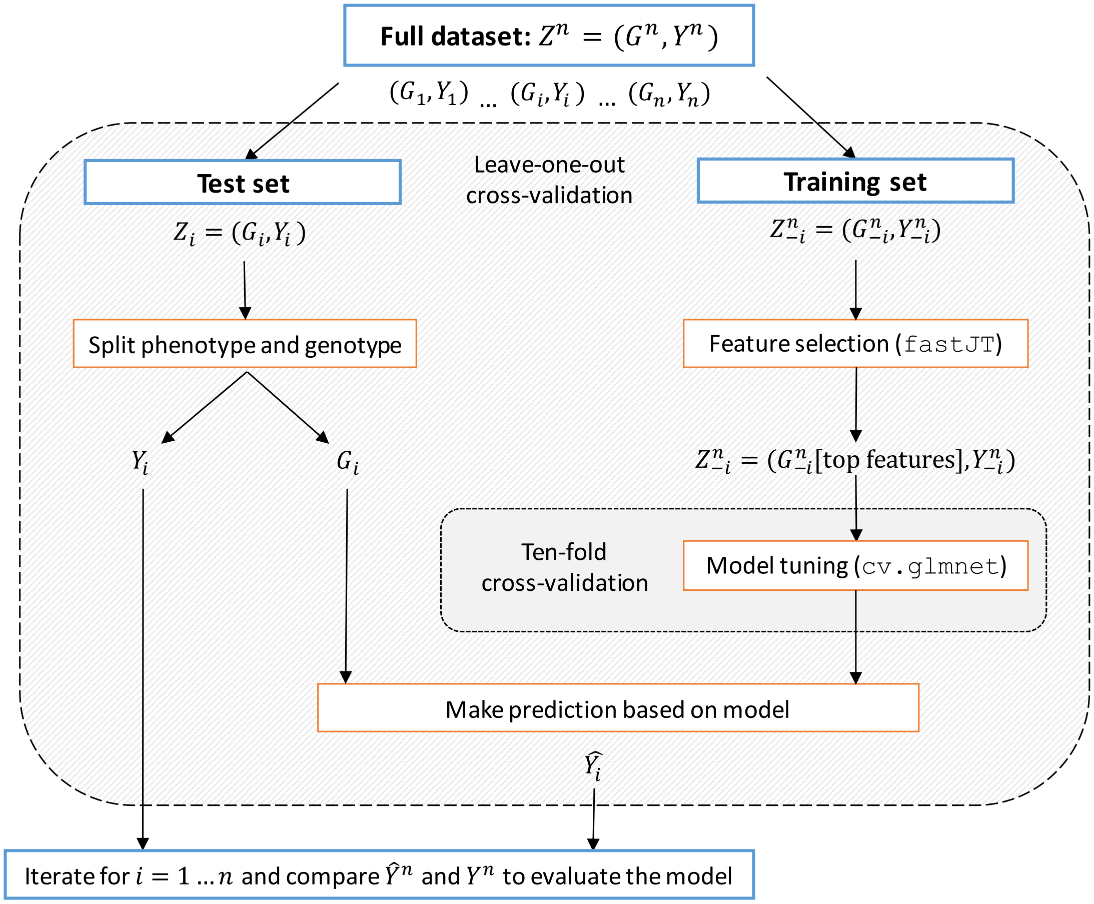

Figure S1: Here $Z^n = (G^n, Y^n)$ represents the feature (G^n) and phenotype (Y^n) data for all n samples. $Z^n_{-i} = (G^n_{-i}, Y^n_{-i})$ represents these data for $n-1$ samples remaining after excluding the ith sample. $Z_i = (G_i, Y_i)$ represents all the feature and phenotype data for the ith sample. Z^n_{-i} and Z_i constitute the training and testing data sets for the ith iteration of the outer leave-one-out cross-validation layer. The top features are selected by applying `fastJT` to the training data $Z^n_{-i} = (G^n_{-i}, Y^n_{-i})$. On the basis of these selected features and the training data, an elastic net model is tuned using ten-fold cross-validation with `cv.glmnet`. The tuned model is applied to G_i, the feature data of sample i, to obtain a prediction $\hat{Y}_i$. This process is repeated for samples $i = 1 \dots n$ to get $\hat{Y}^n = (\hat{Y}_1, \dots, \hat{Y}_n)$. The model is evaluated by comparing the n cross-validated predictions to the corresponding observed values $Y^n = (Y_1, \dots, Y_n)$.
